# Transcriptome analysis of *Haloquadratum walsbyi*: vanity is but the surface

**DOI:** 10.1186/s12864-017-3892-2

**Published:** 2017-07-03

**Authors:** Henk Bolhuis, Ana Belén Martín-Cuadrado, Riccardo Rosselli, Lejla Pašić, Francisco Rodriguez-Valera

**Affiliations:** 1 Department of Marine Microbiology and Biogeochemistry, Royal Netherlands Institute for Sea Research (NOIZ) and Utrecht University, Den Hoorn, the Netherlands; 2 Evolutionary Genomics Group, Departamento de Producción Vegetal y Microbiología, Universidad Miguel Hernández, San Juan de Alicante, Alicante, Spain; 3 Faculty of Medicine, University Sarajevo School of Science and Technology, Sarajevo, Bosnia and Herzegovina

**Keywords:** *Haloquadratum*, Halophile, Transcriptome, Glycoprotein, Bacteriorhodopsin, Archaea

## Abstract

**Background:**

*Haloquadratum walsbyi* dominates saturated thalassic lakes worldwide where they can constitute up to 80-90% of the total prokaryotic community. Despite the abundance of the enigmatic square-flattened cells, only 7 isolates are currently known with 2 genomes fully sequenced and annotated due to difficulties to grow them under laboratory conditions. We have performed a transcriptomic analysis of one of these isolates, the Spanish strain HBSQ001 in order to investigate gene transcription under light and dark conditions.

**Results:**

Despite a potential advantage for light as additional source of energy, no significant differences were found between light and dark expressed genes. Constitutive high gene expression was observed in genes encoding surface glycoproteins, light mediated proton pumping by bacteriorhodopsin, several nutrient uptake systems, buoyancy and storage of excess carbon. Two low expressed regions of the genome were characterized by a lower codon adaptation index, low GC content and high incidence of hypothetical genes.

**Conclusions:**

Under the extant cultivation conditions, the square hyperhalophile devoted most of its transcriptome towards processes maintaining cell integrity and exploiting solar energy. Surface glycoproteins are essential for maintaining the large surface to volume ratio that facilitates light and organic nutrient harvesting whereas constitutive expression of bacteriorhodopsin warrants an immediate source of energy when light becomes available.

**Electronic supplementary material:**

The online version of this article (doi:10.1186/s12864-017-3892-2) contains supplementary material, which is available to authorized users.

## Background

The halophilic archaeon *Haloquadratum walsbyi* dominates sodium chloride (NaCl) saturated thalassic lakes worldwide (up to 10^8^ cells/ml) [1, 2] and may be amongst the more abundant organisms in nature. This microbe is unique due to its peculiar flattened and square shape. After its initial discovery by Anthony E. Walsby in 1980 [3], the organism could not be cultivated for almost 25 years, until 2004 when two strains were isolated and described and their genomes were sequenced. In addition to the Spanish isolate HBSQ001 [4, 5] and the *H. walsbyi* type strain, the Australian strain C23 [6, 7], a further 5 strains were isolated (M. Dyall-Smith, personal communication) and two partial genomes were assembled from metagenome datasets [8]. *H. walsbyi* HBSQ001, is a true halophile that under laboratory conditions grows optimally at a doubling time of about 10 days in near saturated NaCl media (3.3 M) with high concentrations of MgCl_2_ (2 M) [4, 6]. In natural brines, *H. walsbyi* succeeds as a *K*-strategist, species with overall lower energy demands that are capable of efficiently utilizing a vast number of scarce resources [9, 10], in contrast to *R*-strategists, that are mainly fast-growing organisms adapted for high resource utilization. The essential resources for *H. walsbyi* are mainly provided by the main primary producer in hypersaline ecosystems, the halophilic microalgae *Dunaliella salina* [11].

The genome sequence of *H. walsbyi* HBSQ001 yielded several interesting and unique features that were not found in other haloarchaea or any other archaea in general [5]. Unique for haloarchaea is its relatively low GC content (47.9%), with values between 60% and 65% for most other haloarchaea [12]. Amongst others, the genome encoded several extracellular glycoproteins including the extremely large halomucin, an exo-glycoprotein of 9159 amino acids that is probably involved in protecting the cell against dehydration and phage attack [13], and several proteins involved in phosphate and phosphonate metabolism. Unique amongst prokaryotes in general is the extreme low protein coding gene density (76.5%), a value that normally lies between 85%–90% in prokaryotes [14]. *H. walsbyi* is capable of phototrophic growth which is facilitated by a conserved haloarchaeal type bacteriorhodopsin (BopI) that functions as a light driven proton pump [15], generating a transmembrane proton gradient that can be used to drive ATP synthesis via the membrane ATP synthase and to drive other transport processes [16]. A second bacteriorhodopsin coding homolog, *bopII,* is a phylogenetic deep-rooting gene, meaning that it is evolutionary closely related to the common ancestor of currently known *bop* genes although its function is yet to be determined. The genome sequence also revealed genes encoding the two dominant types of intracellular structures: gas vesicles that allow buoyancy and polyhydroxybutyrate granules that store energy.

What the genome sequence could not explain is one of the most fascinating features of *H. walsbyi*, its square morphology and extreme flatness (about 0.1–0.2 μm) [3, 17, 18]. It was hypothesized that the cells aim to maintain an optimal surface to volume ratio to facilitate the membrane related processes for nutrient uptake and phototrophy [4, 19] which is achieved by an extreme flatness. As a consequence, cell division in these large sheets results in square corners, while maintaining the high surface to volume ratio may be one of the distinctive features that makes *H. walsbyi* the most abundant species in hypersaline ecosystems [19].

To date, only a few halophilic Archaea have been subject to sequence based transcriptome analysis. Initially, micro-array studies were performed to study gene expression in *Halobacterium* sp. NRC-1 revealing high responsiveness to its environment, especially to changes in salt conditions [20]. A micro-array based comparison between three haloarchaea, *Halorubrum lacusprofundi*, *Haloferax volcanii* and *Halobacterium* sp. NRC-1 examined growth at increased acidity or alkalinity, and revealed a response in regulating stress, motility, and ABC transporter expression as is seen in prokaryotes in general [21]. *H. volcanii* and *Halobacterium* sp. NRC-1 were studied with a strong focus on the analysis of non-coding small RNA (nc-sRNA) [22] which confirmed that haloarchaeal RNA-based regulation plays an important role in gene transcription. Recently, a transcriptome analysis of the halophilic archaeon *Halolamina* sp. YKT1, isolated from a salt mine in Turkey, was published [23]. The authors compared gene expression under high and low salt conditions and revealed a large number of genes that were either up or down regulated in order to focus its energy towards maintaining the intracellular osmotic balance while minimizing the production of nucleic acids and peptides. High-throughput RNA sequencing was also performed on two closely related strains from the hyper-halophilic bacterium *Salinibacter ruber* that were grown either separately or in co-culture [24]. Transcriptomic patterns from pure cultures were very similar for both strains with the exception of some genes involved in environmental sensing. In co-culture, a small but significant change in their individual transcription patterns was found as compared to those in pure culture showing that both strains reacted in a strain-specific fashion to the presence of the other at the transcriptional level [24].

To better understand the physiology of *H. walsbyi* HBSQ001 and the possible role of its photoheterotrophic growth, we conducted a transcriptome analysis comparing the transcripts of cells harvested from batch cultures at the end of the exponential phase in the light and 12 h later in the dark. Here, we show that light or dark conditions do not significantly affect the expression patterns and that most of the highly expressed genes are related to the expression of exo-glycoproteins, light energy conversion, gas vesicle production and polyhydroxybutyrate (PHB) synthesis.

## Results

### General statistics

RNA sequencing of end exponential phase, dark and light extracted *H. walsbyi* samples (Additional file 1: Figure S1) yielded 111,441,003 and 115,537,266 paired reads, respectively, (Table 1) with an average GC content of 55%. The majority of these reads mapped to the two ribosomal RNA operons (79.3% and 78.2% for the dark and light samples). The dark and light transcriptome contained respectively 2.9% and 3.4% reads that mapped to coding sequences (CDS); while 16.5% and 17.2% of the reads mapped to intergenic regions. Nearly 1% and 0.3% of the sequence reads mapped to two other non-coding RNA features; RNAseP and SRP-RNA (signal recognition particle 7S RNA). Most of the tRNA’s and other small genetic features (<150 bp) could not be mapped due to the larger inserts (> ~ 200 bp) of cDNA fragments required for PE100 sequencing. The average number of transcripts per million (TPM) [25] for the coding region (excluding rRNA and other non-coding RNA features) was 21.3 TPM in the dark and 23.4 TPM in the light (Table 1).

**Table 1:** *H. walsbyi* HBSQ001 general RNA sequencing statistics

	Growth Conditions	
	Dark	Light
Chromosome length (nt)	3,132,794	
Total number of PE100 reads mapped to chromosome	111,441,003	115,537,266
Reads mapped to ribosomal operons	88,414,038	90,353,332
Reads mapped to CDS	3,272,328	3,925,912
Percent reads mapped to CDS	2.9%	3.4%
Percent reads mapped to ribosomal operons	79.3%	78.2%
Percent reads mapped to Intergenic regions	16.5%	17.2%
Percent reads mapped to UTR’s	15.1%	15.2%
Percent reads mapped to non UTR intergenic region	1.5%	2.0%
Percent reads mapped to SRP RNA	0.31%	0.28%
Percent reads mapped to RNAseP	0.88%	0.95%
Average coverage per nucleotide	199×	245×
Median number of reads per CDS	370	430
Average number of reads per CDS	1155	1386
Median TPM per CDS	8.8	9.9
Average TPM per CDS	21.3	23.4
Percentage of CDS expressed (>Percentile 10 (less than 37 and 43 reads)	17.6%	17.2%
Percentage of CDS expressed (>0 reads) (6 CDS)	94.6%	94.9%
GC	55%	54%

CDS, coding DNA sequence; IR, intergenic region

### Gene expression comparison between light and dark

**Fig. 1:**
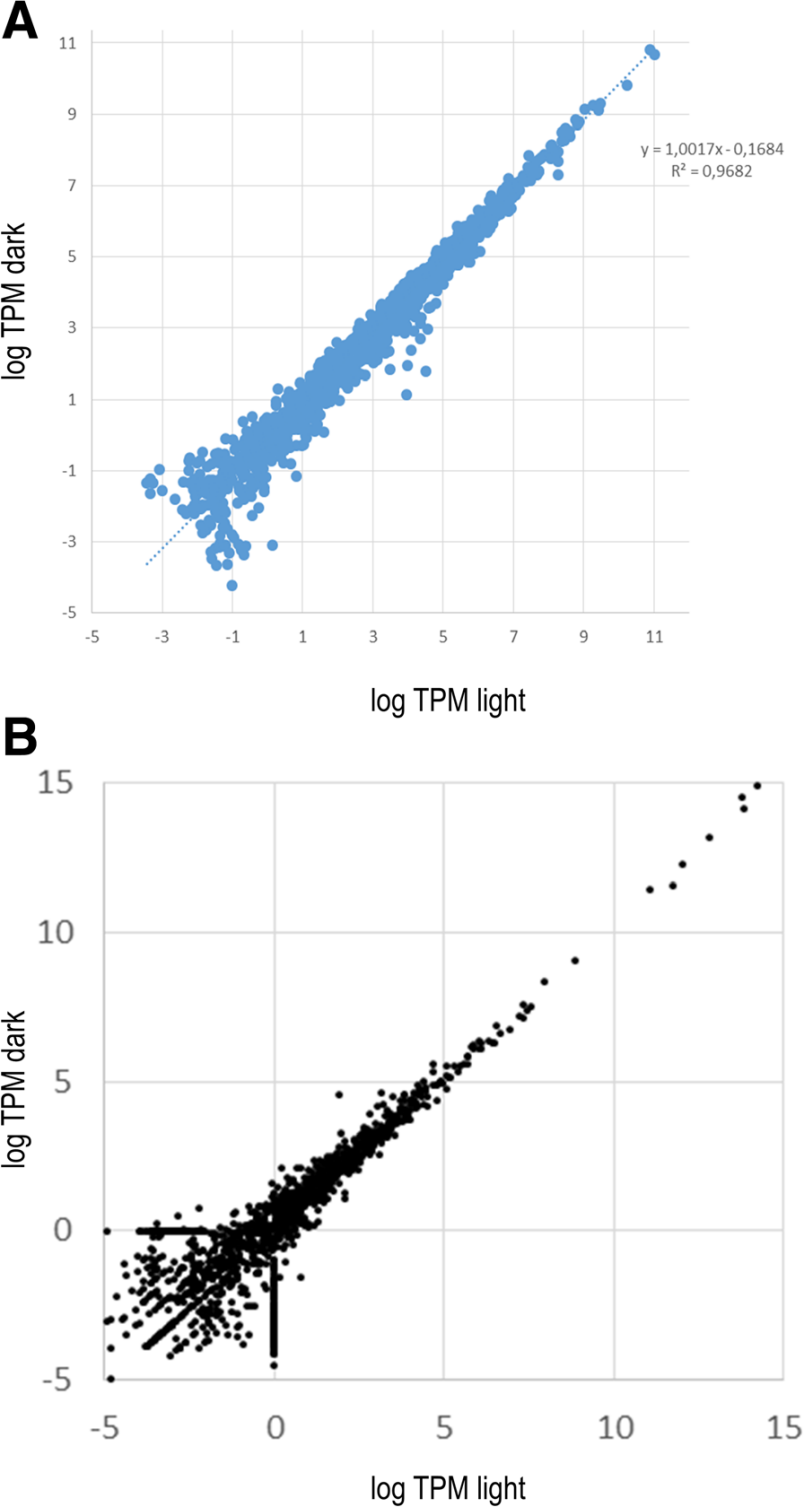
Comparison of gene expression between the *light* and *dark* sample for the protein coding sequences (**a**) and intergenic regions (**b**). The expression levels in TPM were log transformed

**Fig. 2:**
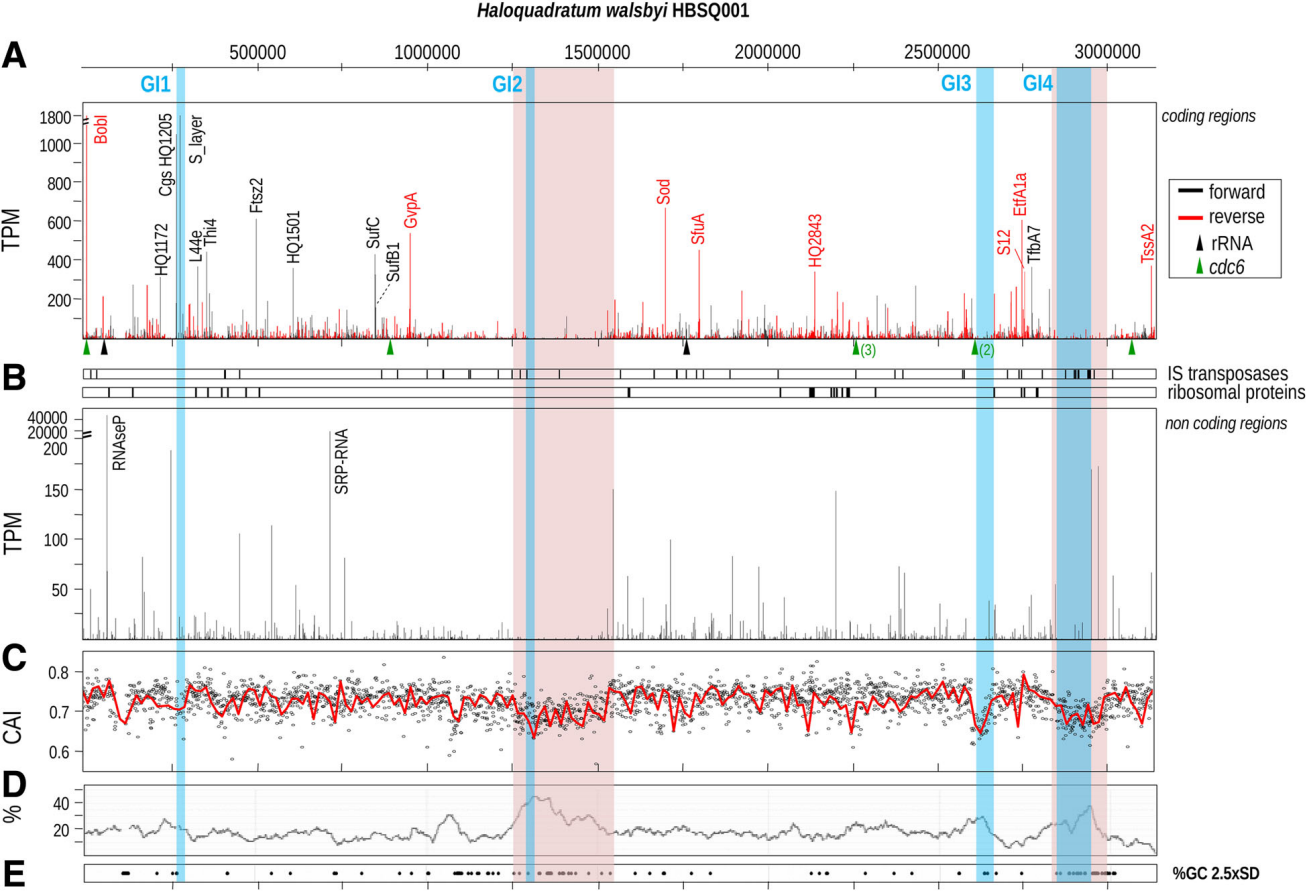
Levels of transcription (TPM) of the coding (**a**) and non-coding regions (**b**) of *H. walsbyi* HBSQ001. Positions for the rRNA operons, cdc6 genes, IS elements and genes encoding ribosomal proteins are indicated (see legend). Only the products (protein or RNA) encoded by the genes with TPM > 300 are indicated. **c** Codon adaptation index (CAI) for each of the genes. **d** Percentage of the hypothetical proteins in a window of 100 and **e** regions with a GC-content deviation bigger than 2.5 x SD. The *blue bands* indicate the position of the ribosomal RNA gene clusters. The two *pink bands* indicate the two low expressed regions in the genome

Comparison of the light and dark sample revealed no significant differences (i.e. no expression differences >2.5 standard deviation) between the two datasets for both coding sequences and intergenic regions (Fig. 1a and b). Due to the lack of differential gene expression between the light and dark samples we choose to average the expression data obtained from both samples for further analysis. Plotting expression levels per CDS or intergenic region along the genome revealed a clearly uneven distribution of high and low expressed genes (Fig. 2a and b). When comparing the first with the second half of the genome, the average transcription rates (in TPM) were similar (54 vs 46%). However, when we divided the genome in 4 equal parts, the distribution of highly expressed genes in respective quarters was Q1 - 53%, Q2 - 1%, Q3 - 44% and Q4 - 2%, revealing two highly expressed genomic regions and two poorly expressed regions. Since this distribution could be caused solely by the 30 highest expressed genes (>200 TPM) we removed these from the calculation but still showing that overall expression levels in the first and third quarter were higher than in the other two quarters, although the differences were much less obvious (Q1 - 36%, Q2 - 15%, Q3 - 25% and Q4 - 24%, respectively).

### Abundantly and lowly expressed genes

**Fig. 3:**
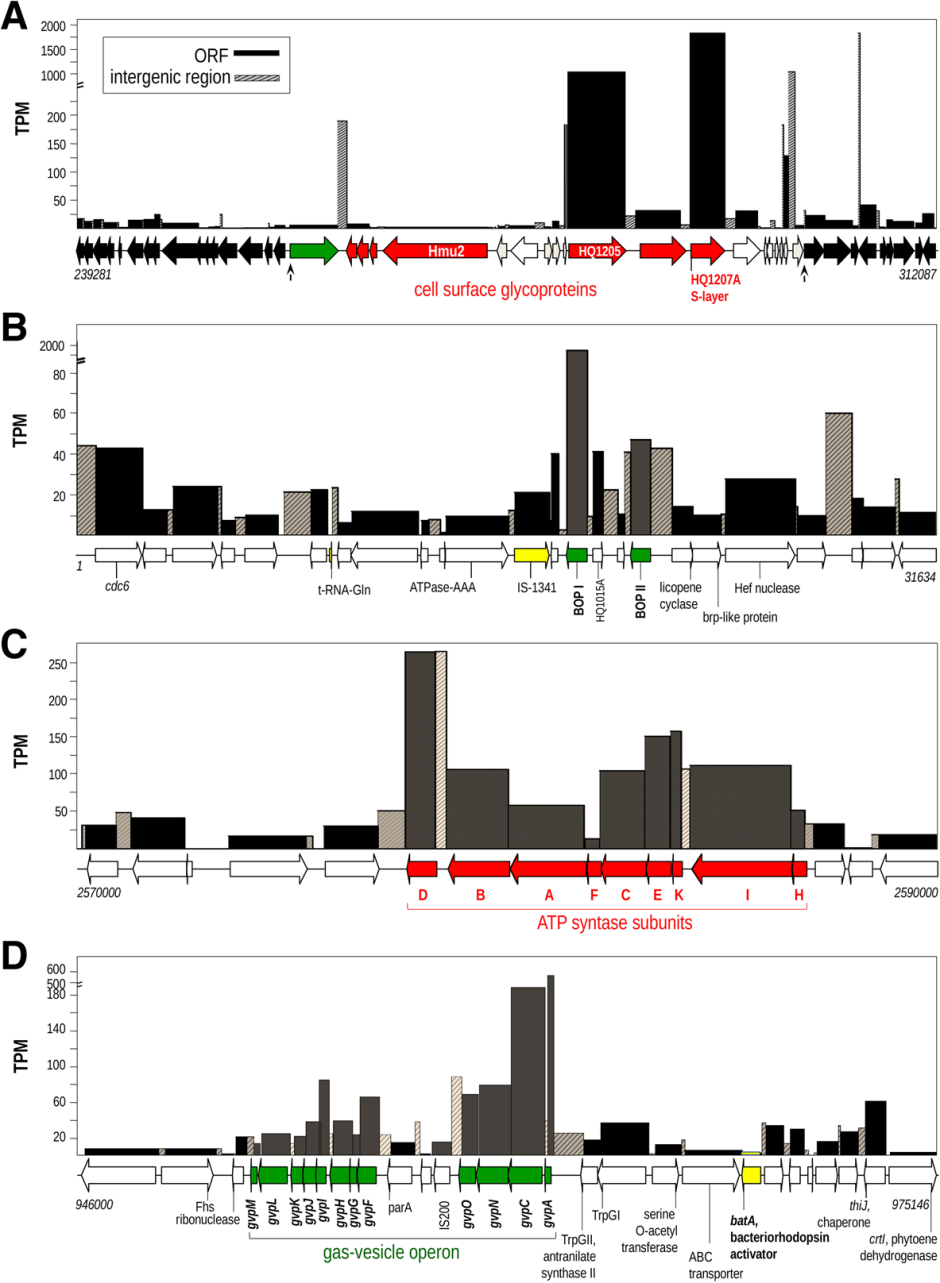
Expression of regions of interest. (**a**) Genomic island 1, *Arrows* indicated the beginning and the end of GI1 accordingly to Martin-Cuadrado et al. 2015 [26]. (**b**) Bacterioopsin genes *bopI* and *bopII*. (**c**) ATP synthase gene cluster and (**d**) the gas vesicle synthesis operon

Thirty non-clustered protein coding genes were expressed at more than 10 times the average (Table 2). The three most highly expressed genes were the *csg* gene (HQ1207A) at 1843 TPM, encoding a 988 amino acid long S-layer glycoprotein gene (Fig. 3a), directly followed by *bopI* (HQ1014A) at 1833 TPM, encoding the proton pumping bacteriorhodopsin (Fig. 3b), and HQ1205A at 1050 TPM, a gene encoding a 1627 amino acids long cell surface glycoprotein that is probably a component of the cell wall together with *csg* [26] (Fig. 3a). Other highly expressed genes encoded proteins involved in oxygen radical stress response (superoxide dismutase; HQ2461A and peroxiredoxin *bcp4*; HQ3020A) or cell division (*ftsZ2*; HQ1415A, *ftsZ1*; HQ1243A and *sepF*; HQ3071A).

**Table 2:** Highest expressed genes with expression levels (TPM) more than 10 times the average per coding sequence, sorted in order of expression level

Gene-designation	gene	TPM	Function
HQ1207A	*csg*	1843	Cell surface glycoprotein
HQ1014A	*bopI*	1833	Energy conversion
HQ1205A	HQ1205A	1050	Cell surface glycoprotein/ adhesin
HQ2461A	*sod*	671	Superoxide dismutase
HQ1415A	*ftsZ2*	615	Cell division protein
HQ3385A	*tef1a*	610	Elongation factor 1-alpha
HQ1782A	*gvpA*	542	Gas vesicle production
HQ2545A	*sfuA*	456	Iron transport
HQ1276A	*thi4*	447	Putative thiamine biosynthetic enzyme
HQ1706A	*sufC*	432	Fe-S cluster assembly ATPase
HQ3729A	*tssA2*	374	Sulfurtransferase
HQ1253A	*rpl42e*	370	50S ribosomal protein L44e
HQ3408A	*tfbA7*	369	Transcription initiation factor IIB
HQ1501A	HQ1501A	363	Uncharacterized protein
HQ3391A	*rps12*	346	30S ribosomal protein S12
HQ2843A	HQ2843A	346	Uncharacterized protein -DUF171 family
HQ1707A	*sufB1*	331	Fe-S cluster assembly
HQ1172A	HQ1172A	318	RNA binding / TRAM domain protein
HQ1100A	HQ1100A	279	Uncharacterized protein
HQ1133A	HQ1133A	276	Uncharacterized protein
HQ3117A	*citB*	274	Aconitate hydratase (TCA cycle enzyme)
HQ3366A	HQ3366A	269	Uncharacterized protein
HQ3457A	*fer7*	258	Ferredoxin (2Fe-2S) - electron transfer
HQ2649A	HQ2649A	247	Uncharacterized protein -DUF293 domain
HQ3356A	*porA*	243	pyruvate: ferredoxin oxidoreductase
HQ2902A	*rpl10*	243	50S ribosomal protein L10
HQ1283A	*rpl43e*	235	50S ribosomal protein L43
HQ3242A	*atpD*	234	V-type ATP synthase subunit D
HQ3309A	*rps8e*	232	30S ribosomal protein S8e
HQ3020A	*bcp4*	223	Peroxiredoxin

The second bacterioopsin gene, *bopII* had an expression level slightly above average but much lower than *bopI* under the conditions studied (Fig. 3b). In contrast, the expression of genes *bat* (bacterioopsin activator) and *bap* (bacterioopsin accessory protein), both considered to be essential for rhodopsin function [27], was almost two times higher than average (Additional file 2: Figure S2). As expected, amongst the 214 genes that are expressed at more than 5 times average, 21 encode for ribosomal proteins and 16 encode for proteins involved in transcription and translation initiation and regulation (Additional file 3: Table S1 Sheet 1).

A number of highly expressed genes were found to be part of larger gene clusters. Nearly all genes in the V-type ATP synthase coding cluster (*atpDBAFCEKIH*) were highly expressed (Fig. 3C). The most highly expressed gene within this cluster was *atpD* which, together with poorly expressed *atpF* encodes the central rotor axle of the ATPase [28]. The gas vesicle producing *gvp* cluster was also highly expressed, especially the major structural protein coding gene *gvpA* (~25 times average) and, to a lesser extent, *gvpC* (~5 times average) (Fig. 3D). The other genes in this cluster were expressed below (*gvpMLKJG*) or slightly above (*gvpIHFON*) average. Within the cluster involved in PHB synthesis, two hypothetical protein coding genes (HQ2312A and HQ2313A) were expressed 5 to 10-fold higher than average, whereas *phaB* encoding the NADPH-dependent acetoacetyl-CoA reductase and *phaC* encoding the PHB synthase were expressed at an average level (Additional file 3: Table S1 Sheet 2). The gene *moaC*, which encodes an enoyl-CoA hydratase involved in supplying (R)-3-hydroxyacyl-CoA from the beta-oxidation pathway to the PHB biosynthetic pathway, was also highly expressed (7-fold average). Another cluster, whose genes showed above average expression was *nuo* cluster that encodes the proton-translocating NADH dehydrogenase. In particular higher expression was observed with genes *nuoA*, *nuoB* and *nuoCD* that encode the structural subunits of the dehydrogenase complex (Additional file 3: Table S1 Sheet 2). Of the 58 ribosomal proteins identified in the transcriptome, 22 were expressed more than 2 times average and 9 were expressed more than 5 time average (Additional file 3: Table S1 Sheet 3). The genes encoding the TCA cycle proteins are located at different regions of the genome but are all expressed above average (Additional file 3: Table S1 Sheet 4).

The transcriptome contained transcripts of 221 transport protein coding genes, 34 of which were expressed above average, with the relative expression of 17 genes exceeding the average at least 2 times (Additional file 3: Table S1 Sheet 5). Fifteen of these genes encoded ABC-type transporters that depend on ATP as driving force. The other two genes encoded a putative drug/metabolite transporter (DMT superfamily) and a substrate binding protein of a TRAP transporter gene cluster (HQ1442A-HQ1444A) whose substrate is currently unknown. In total, 7 of these 17 transport related genes encoded substrate binding proteins. The highest expressed gene cluster amongst these encodes an iron transport protein complex SfuABC of which the substrate binding protein coding gene, *sfuA* (HQ2545A), is amongst the highest expressed genes overall (Table 2). Genes encoding another putative ABC-type transport system is also involved in iron scavenging and Fe-S cluster assembly [29] (*sufAB1B2*; HQ1706A-HQ1708A). Two other highly expressed genes encoding transporters are involved glutamine./glutamate (*glnH* – Hq2732A) and in copper uptake (*nosY* –HQ1143A). Finally, *H. walsbyi* HBSQ001 contains 7 gene clusters encoding ABC-type branched-chain amino acid transport systems, each consisting of 5 genes (*livFGHJM1-7*). In 5 of these *liv* clusters only the substrate binding protein coding gene *livJ* is expressed above average.

In the cultures, that were supplemented with glycerol and sodium pyruvate as carbon and energy source, a higher than average expression was found for several genes involved in glycerol metabolism (Additional file 3: Table S1 Sheet 6). The glycerol kinase gene (*glpK*: HQ1733A) was nearly 5-fold higher expressed than average. Its neighboring gene HQ1732A that expressed a putative protein with unknown function is polycistronic transcribed at a more than 2.5 fold higher level than average. More than 2 fold higher expression than average was also found for the alpha subunit of glycerol-3-phosphate dehydrogenase encoded by the *glpA* gene that belongs to the *glpABC* gene cluster and is located directly downstream of *glpK*. A second *glpA* gene located directly downstream of the cluster *dhaKLM* (which encodes a low expressed phosphotransferase (PTS) dependent dihydroxyacetone uptake system) was also expressed above average. Methylglyoxal synthase encoded by *mgsA* (HQ1527A) that converts dihydroxyacetone phosphate to methylglyoxal and phosphate was also highly expressed (107 TPM).

Notably, a significant number of genes encoding hypothetical proteins or proteins with yet unknown function were above average to highly expressed. These include 17% of the hypothetical protein coding genes expressed at more than 10 fold average and 20% of these genes expressed at more than 5 fold average.

Nearly 5% (127) of the annotated genes were not expressed at all and 15% (417) had on average less than one transcript per million of which 95% were annotated as hypothetical proteins (Additional file 3: Table S1 Sheet 7). The only unexpressed gene with clear annotation encodes for an L-lactate permease (HQ2551A). The highest density of low- to non-expressed genes was found at the end of the first (from position 1.250.000 to 1.530.000) and second half of the genome (from position 2.850.000 to 3.030.000) (Fig. 2). These low expressed regions are characterized by an on average lower codon adaptation index (CAI) (Fig. 2c), higher GC content (Fig. 2d) and were rich in hypothetical genes for which it is yet unclear whether they are translated to proteins under any condition (Fig. 2c and d). Of the previously identified genomic islands (GI’s), two including the largest (GI4) overlap with the low expressed regions (Fig. 2). Amongst the very low expressed genes were *hmu* (3.3 TPM) (Additional file 2: Figure S2), the gene that encodes the giant halomucin protein (9159 amino acids) as well as the two smaller halomucin genes *hmu2* (2.7 TPM) and *hmu3* (5.1 TPM). We also analyzed the genes encoded on the plasmid PL47, however only two of the plasmid encoded genes, HQ4010A and HQ4030A were significantly expressed and both encode hypothetical proteins with unknown function.

### Expression patterns in functional groups

**Fig. 4:**
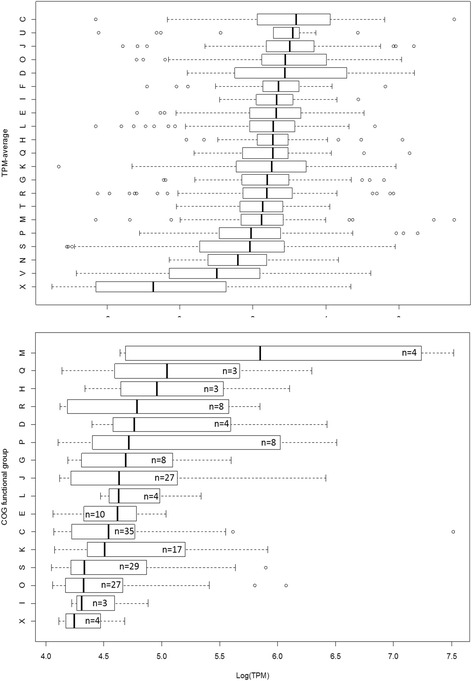
Median gene expression ranked by COG functional groups of the total protein coding dataset (**a**) and of the top 200 highest expressed genes (**b**)

Expressed genes were classified in functional groups according to the arCOG database [30] (Additional file 3: Table S1 Sheet 8). The distribution of median expression levels within these groups is presented as a boxplot of the log transformed expression values comprising all protein coding genes present in the genome including those that are not expressed (Fig. 4a). Median expression is highest in genes involved in energy production and conversion (arCOG group C) followed by intracellular trafficking, secretion, and vesicular transport (arCOG group U) - translation, ribosomal structure and biogenesis (arCOG group J) - posttranslational modification, protein turnover, chaperones (arCOG group O) and cell cycle control, cell division or chromosome partitioning (arCOG group D). When only considering the top 200 highly expressed genes with expression levels larger than 2.5 times the average and more than 2 representatives per functional group, the highest median values are found for genes encoding proteins related to cell wall/membrane/envelope biogenesis (arCOG group M), secondary metabolites biosynthesis, transport and catabolism (arCOG group Q), coenzyme transport and metabolism (arCOG group H), general function prediction (arCOG group R) and cell cycle control, cell division, chromosome partitioning (Fig. 4B). Overall the median expression level for energy related processes in the top 200 is low with the exception of one outlier, the above mentioned gene *bopI*.

### Small RNA’s and other genome features

Putative small non coding RNA’s (snRNA), some of which were previously unannotated in the genome, could be identified in the transcriptome data. After excluding ribosomal RNA genes and putative snRNA genes that overlapped with previously annotated coding regions, 147 putative snRNA genes could be detected within the *H. walsbyi* chromosome plus 6 on the plasmid PL47. The identified snRNA genes were clustered into four categories crRNA, ncrNA, snoRNAs and tRNA genes. CRISPR (clustered regularly interspaced short palindromic repeats) RNAs (crRNA) are RNA molecules that provide protection against phage and plasmid infection. *H. walsbyi* HBSQ001 contains one CRISPR region composed of five spacers flanked by seven direct repeats of 25 bp while putative Cas proteins were not identified in this strain. In addition, the CRISPR region is interrupted by a putative insertion sequence element. Nevertheless, the CRISPR region is significantly expressed. *H. walsbyi* HBSQ001 expresses 79 non-coding RNA (ncRNA) that range in size from 30 to 495 bp. The levels of expression of two of these ncRNAs are surpassed only by the ribosomal rRNA genes. These are the 7SL RNA, which has a signal recognition function to guide membrane proteins to the protein translocation complex and the ribozyme RNase P. Other high expressed ncRNAs were SECIS (selenocysteine insertion sequence), involved in the translation of UGA codons as selenocysteines and HgcC (“high GC” family RNA) with unknown function. Small nucleolar RNAs (snoRNA) are well-characterized small RNAs that play a role in the nucleus of eukaryotes but are also found in archaea. Two classes of snoRNAs have been characterized that are involved in the modification of rRNA, C/D-box RNAs, which guide methylation sites in rRNAs, and H/ACAs RNAs involved in pseudouridine formation. In *H. walsbyi* HBSQ001, 24 C/D-box RNAs are found in the major chromosome and 6 on the plasmid and had variable expression levels. H/ACAs RNAs were not identified. Most tRNAs were lost in the sequencing processing with the exception of the tRNA-Trp, the largest tRNA with 178 bp compared to the approximate 75 bp of other tRNAs. Putative circular cRNA molecules were not detected.

## Discussion

### No differential gene expression between light and dark period

Although Archaea are generally not known for having an endogenous molecular clock that may regulate gene expression in a circadian rhythm, *H. walsbyi* is a photoheterotrophic organism that may benefit from differential gene expression during the light or dark period in putting less energy towards the synthesis of light dependent proteins such as both bacteriorhodopsins and halorhodopsin, a light driven chloride transporter. Similar mechanisms are well described for Cyanobacteria in which a controlled sequence of phosphorylation and dephosphorylation of the key circadian clock protein (KaiC) is responsible for the regulation of gene transcription of a large number of genes [31]. However, outside the Cyanobacteria no circadian regulation is observed in prokaryotes. When comparing the transcriptome from the light and dark sample, no significant difference (expression differences >2.5 standard deviation) in gene expression was observed throughout the genome. We can therefore conclude that gene expression in *H. walsbyi* is not regulated by a circadian like mechanism that switches gene expression patterns within 12 h nor does it appear to be regulated by light or darkness as physiological cues. However, due to the lack of significant differences the two samples could now be considered as a biological replicate from which we used the mean values to analyze genome wide gene expression patterns. Existence of putative circadian clock like mechanism can however not be ruled out amongst other haloarchaea. Microarray analysis of gene transcripts in *Halobacterium* NRC-1 grown under a 12 h light / 12 h dark regime did reveal significant diurnal oscillatory transcription in up to 12% of its genes including free-running behavior after the light dark stimulus is stopped [32].

### Energetics

Despite the absence of a light/dark differential regulation, light is an important driver in energy generation given the high expression of *bopI*, the second highest expressed gene overall. In the light, bacteriorhodopsin is involved in the generation of a proton gradient which in turn can be used to drive a multitude of proton dependent transmembrane transport processes. Why *bopI* expression is high even in the dark is unknown. Potentially there is a high turnover of this protein although that would not be in accordance with the highly stability and stress resistance characteristics of bacteriorhodopsins in other Halobacteria that in fact are amongst the most stable proteins known to date [33]. High expression of rhodopsins is also found in *H. salinarum* strain R1 under both light and dark conditions [34] and in the bacterium *S. ruber* that expresses a xanthorhodopsin gene encoding a homologous light dependent proton pump [24]. Also the green light absorbing proteorhodopsin (PR) of the flavobacterium *Dokdonia donghaensis* PRO95 expressed its PR gene both in the light and in the dark even though light was not found to stimulate growth [35]. Several *H. walsbyi* genes are identified to encode putative regulators of *bopI* expression; a small zinc finger protein, HQ1083B, and a small basic protein, Bp (HQ1083A). Both cooperate with Bat proteins to activate *bopI* transcription under phototrophic growth conditions in *H. salinarum* R1. HQ1083A and *bat* were both expressed above average and therefor explain the high expression of *bopI*. However, the physiological significance of high *bopI* expression especially in the dark remains unclear. A second zinc finger protein with unknown function, HQ1015A that is divergently transcribed directly upstream of *bopI* is also highly expressed. Based on analogy with a homologous protein in *H. salinarum*, HQ1015A is likely also involved in the regulation of *bopI* transcription [36]. The second bacterioopsin coding gene *bopII* that is located within three genes from *bopI* is expressed above average. This phylogenetic deeply rooting protein may not function as a light driven proton pump but instead may have a sensory function, although motility in *Haloquadratum* is apparently limited to controlling buoyancy via the gas vesicles. The expression of the haloopsin gene is below average but this is apparently sufficient to fulfil its role as light driven chloride pump. Proper function of the opsin proteins is dependent on the retinal chromophore of which a key enzyme, phytoene synthetase, encoding gene *ctrB* (HQ2860A) is expressed at nearly 3 fold average.

The light generated proton gradient can be efficiently used by several proton translocating protein complexes such as the proton dependent ATPase, generating the universal energy storage molecule ATP, and it can drive the reverse process of the proton translocating NADH dehydrogenase. Genes encoding both complexes are highly expressed underlining the need for ATP and reducing equivalents such as NADH. Like other photo-heterotrophic prokaryotes, *Haloquadratum* could use the surplus in light generated proton gradient via a reverse electron transport mechanisms through the NADH dehydrogenase that reduces NAD+ to NADH in the process. Together these processes allow for optimal and efficient use of the light generated proton gradient.

In the HAS growth medium and in the natural ecosystem, the main organic carbon and energy source is glycerol, not surprisingly a whole suit of transcripts for the enzymes involved in glycerol metabolism, glycerol kinase (GlpK) and glycerol-3P dehydrogenase (GlpA), were highly expressed. This includes HQ1732A the gene encoding an uncharacterized putative membrane protein with 7 predicted transmembrane helices located immediately upstream of *glpK.* Homologs of the HQ1732A gene product are found in other haloarchaea with between 6 and 8 transmembrane helices and in all instances in the string database (https://string-db.org/) found in close association with glycerol kinase and glycerol-3P dehydrogenase. Potentially this protein and its homologs may play an essential role in glycerol metabolism or even glycerol uptake. Low expression of genes involved in the unique archaeal phosphotransferase uptake system involved in DHA metabolism possible reflects the absence of DHA or a DHA producer like *Salinibacter ruber* [37] in the medium used.

### Patterns in genome wide expression levels

A significant percentage of the dark (16.5%) and light (17.2%) reads mapped to intergenic regions of which approximately 90% can be attributed to potential untranslated but transcribed regions (UTRs). Expression levels of these UTRs can be several tens of folds higher than the actual coding sequence. Although both 5’and 3′ UTRs play a significant role in gene expression [38], we cannot simply translate and compare UTR expression levels and infer actual protein synthesis rates. Therefor we only focused on the expression of the protein coding sequence itself for comparison.

Two highly expressed regions were identified on the genome as well as two poorly expressed regions. The high and low expressed regions are found at the beginning and ends of the two halves of the genome. Commonly prokaryotes have a single origin of replication with most of the housekeeping genes and the ribosomal RNA gene cluster (5S, 16S and 23S rRNA) located near the origin [39]. Moreover, transcription levels are also higher near the origin or replication [40]. *H. walsbyi* encodes two rRNA gene clusters and high expression levels are centered on both clusters. The reason for the high expression of genes in the region of the second rRNA cluster, far from the origin of replication is unknown. Potentially it reflects an ancient genome duplication event with a remnant or independent second origin of replication, a feature not uncommon in Archaea, given the detection of three origins of replication in *Sulfolobus* species [41, 42]. However, *cdc*6-like DNA replication protein coding genes, like the one associated with the established origin of replication in *H. walsbyi* are not found in close proximity of the second 16S rRNA cluster. Moreover, several typical housekeeping genes like those involved in the TCA cycle are not located near the origin but rather in the second half of the genome near the second rRNA gene cluster and three alternative *cdc6* like DNA replication protein coding genes (*orc4*, *orc6*, *orc7*). The poorly expressed regions at the end of the first and second half of the genome are characterized by an on average higher GC content and are rich in hypothetical genes with unknown function. Potentially this part of the genome is rich in horizontally acquired genes that may serve as a genetic toolbox for future environmental challenges or is only expressed in the natural environment. The only plasmid (PL47) of strain HBSQ001 serves a potential similar role since only two of the 36 genes were expressed under the imposed cultivation conditions.

### Gene expression of a hyperhalophile

The square extreme halophile *H. walsbyi* is in many ways enigmatic. Not only is it capable of maintaining a unique square morphology, it does so at extreme high salinities far above NaCl saturation and is widespread in these hypersaline environments [3, 17–19, 43, 44]. Maintaining membrane integrity and protecting cells against desiccation and other stresses in this hostile environment requires specific adaptations at both genetic and physiological level. This transcriptome analysis clearly reveal the importance of maintaining membrane integrity by two highly expresses genes encoding putative extracellular S-layer glycoproteins in the top three, gene HQ1205A and HQ1207A. Surprisingly, the large, 27,477 nucleotide long halomucin coding gene is expressed below average. Low expression is also observed in the other two halomucin coding genes. Potentially only a few mRNA copies are required to produce sufficient halomucin proteins and to reduce energy and resources needed for the synthesis and translocation of these costly proteins [13]. Moreover, it has been argued that halomucin may also be involved in protection against the abundant halophages present in their natural habitat but absent in pure cultures of *H. walsbyi* HBSQ001, a defense mechanism also found to protect mammalian epithelial cells [45]. High expression of the cell surface glycoproteins HQ1205A (4881 bp) and HQ1207A (2967 bp) is apparently sufficient to protect membrane integrity at the extant cultivation conditions. The gene in between, HQ1206A, encodes a third, 3780 bp long cell surface glycoprotein and is moderately expressed while HQ1204A, directly upstream of this cluster is highly expressed. However, HQ1204A is a much smaller gene (216 bp) and encodes a putative CopG/RHH family DNA binding protein that might be involved in regulation of the adjacent cell surface glycoprotein genes.

Gas vesicles may play an important role in supporting aerobic growth, especially in solar salterns where oxygen dissolution close to the surface is limited and other means of motility like a flagella are lacking in *H. walsbyi*. Alternatively gas vesicles may play a role in aligning the cells with their surface to the sun for optimal generation of the bacteriorhodopsin derived proton gradient. Indeed, *H. walsbyi* invests lots of energy in the production of gas vesicles which is evident from the abundantly present number of gas vesicles in its cells and the high expression levels of gas vesicle genes. High expression is evident in the two major structural protein coding genes *gvpC* and *gvpA*, and to a lesser extent *gvpN* (encoding a chaperone required for the assembly of gas vesicles), *gvpO* (required for gas vesicle synthesis and potential transcription regulator [46]) and *gvpI* (required for termination of GvpA incorporation in the gas vesicle protein complex [47]). In *Halobacterium salinarum* growing under anoxic conditions and in the presence of citrate, the *gvpACNO* genes were found to be significantly overexpressed [48]. Although the *gvp* gene cluster has a similar arrangement as in *H. salinarum* and *Haloferax mediterranei*, two important regulatory genes are absent in *H. walsbyi*. Absence of *gvpE*, a gene encoding a transcriptional activator of GvpA [49] and *gvpD*, encoding a protein involved in the repression of the gas vesicle formation [50], suggests a different regulation mechanism for gas vesicles compared to other haloarchaea. Although it was hypothesized that gas vesicle production in *H. salinarum* was stimulated by citrate rather than by anoxic conditions, *H. walsbyi* was grown in the absence of citrate and up-regulation of the *gvp* genes found in the present study may reflect the sub-oxic conditions in the applied cultivation set up of standing cultures without active aeration by shaking or bubbling with air. However, sub-oxic conditions do not explain the high expression of superoxide dismutase gene (*sod*). Especially aerobic growth comes with the toll of generating high concentration of oxygen radicals and hence the need for a detoxifying SOD protein [51]. However a basic expression of *sod* in *H. cutirubrum* was found under anaerobic conditions while expression was induced in the presence of paraquat, a generator of oxygen radicals [52]. What causes the high expression in *H. walsbyi* under the extant growth conditions is unknown but continuous presence of SOD is essential for reducing cumulative oxidative damage [51].

High expression of the substrate binding protein coding gene (livJ) from 5 of the 7 clusters encoding ABC-type branched-chain amino acid transporters may indicate a potential shortage in amino acids or nitrogen in general. Only low concentrations of amino acids are supplied via yeast extract in HAS medium and may explain the need for high expression of substrate binding protein to scavenge the growth medium for branched amino acids (leucine, isoleucine and valine). Having 7 similar gene clusters in the genome may also reflect the high requirement for amino acids in the natural environment. High concentrations of PHB in Bacteria are associated with a carbon overflow mechanism occurring at a relative excess of carbon relative to a limitation of oxygen, nitrogen, phosphorus, or potassium resulting in the accumulation of reducing power [53]. In haloarchaea like *H. mediterranei*, neither oxygen nor nitrogen but rather phosphate limitation is a strong inducer of PHB accumulation [54]. Free phosphate concentrations are naturally low in crystallizer ponds where *H. walsbyi* dominates due to the low solubility of phosphate at the extant high magnesium concentrations. Indeed, *H. walsbyi* is well equipped with a suit of enzymes directed to scavenge phosphates or phosphonate as alternative source of phosphate [5]. Most likely phosphate is present in sufficient quantities since none of the phosphate or phosphonate transporters are significantly over expressed.

In three of the highlighted operons; NADH-dehydrogenase, Gas-vesicle biosynthesis and ATP-synthase there are several indications for polycistronic gene transcription which is characterized by short intergenic distances between genes or even overlapping genes and an initial high expression at a single 5’UTR followed by a more or less even read distribution over the genes within the transcription unit [55]. The ATP-syntatse gene cluster appears to consist of three transcription units consisting of the polycistronic clusters *atpIH* and *atpKECFABD* and the monocistronic unit consisting of *atpD*. The units are >200 nt apart whereas genes within the unit are overlapping (−3, −13 nt) or have a very short, 2-24 nt overlap (Additional file 4: Figure S3A). The NADH-dehydrogenase encoding cluster reveals two putative polycistronic transcription units consisting of *nuoABC/DHI* and *nuoJ1J2KLMN* (Additional file 4: Figure S3B). Genes within this cluster either overlap with three nucleotides or are 0, 1 or 2 nucleotides apart. In contrast, for the gas vesicle coding *gvp* gene cluster the situation becomes less apparent. In addition to the genes interrupting the gene cluster, the intergenic regions in this gene cluster are overall more spaced (Additional file 4: Figure S3C). Potential polycistronic transcribed units consist of *gvpKJI* and *gvpHGF*. Although *gvpC* appears to be part of a polycistronic unit based on its 3 nucleotide overlap with *gvpN*, its expression and read coverage is significant higher than for *gvpN*, suggesting specific, monocistronic transcriptional control. Together with the highly expressed gene *gvpA*, monosistronic transcription may be essential for the two main structural gas vesicle proteins to achieve the high number of gas vesicles normally observed in the cell cultures.

## Conclusions

Little is known at this moment about gene expression patterns in Archaea in general. The transcriptome analysis of *H. walsbyi* gave insight in a number of highly expressed genes as well as two large regions with very low expression. Gene expression in *H. walsbyi* is all about surface. It devotes a lot of its resources to the expression of genes encoding glycoproteins involved in S-layer biosynthesis and surface protection against desiccation and maintaining the flat square morphology to accommodate a large surface for membrane related processes of energy generation and the uptake of nutrients.

## Methods

### Cultivation

*Haloquadratum walsbyi* strain HBSQ001 (DSM16790) was cultivated in 1 L tissue culture flasks equipped with filter screw caps (TPP - Techno Plastic Products AG, Switzerland) containing 300 ml of HAS medium (composition per litre: 195 g of NaCl, 50 g of MgSO_4_.7H_2_O, 35 g of MgCl_2_.6H_2_O, 5 g of KCl, 0.25 g of NaHCO_3_, 1 g of NaNO_3_, 0.5 g of CaCl_2_.2H_2_O, 0.05 g of KH_2_PO_4_, 0.03 g of NH_4_Cl, 20 ml of Tris-HCl (1 M, pH 7.4) and as carbon and energy source, 0.5 g of glycerol and 1 g of sodium pyruvate [4]). Cells were incubated at 30 °C under a dark/light regime of 16 h light and 8 h dark. Growth was followed spectrophotometrically at 600 nm (Additional file 1: Figure S1).

### RNA extraction

For RNA extraction, 200 ml samples were taken at the end of the exponential growth phase (Additional file 1: Figure S1). The samples were taken at the middle of the light period and exactly 12 h later at the middle of the dark period) and kept in the dark in aluminum foil until cell lysis. Per sample, 200 ml of cell culture was centrifuged in 4 aliquots of 50 ml in TubeSpin® Bioreactor50 tubes (TPP) at 5000 G. Supernatant was carefully discarded and remaining medium was removed from the inside of the tube to prevent the high salinity from interfering with the RNA isolation procedure. RNA was extracted from each of the 8 pellets using the ZR Fungal/Bacterial RNA MiniPrep kit (Zymo Research, Irvine, USA) following the manufactures recommendations and eluted in 100 μl of elution buffer. After RNA extraction the 4 samples were pooled resulting in one night and one day sample with an average RNA yield of 300 ng/μl and a total yield of 120 μg. Contaminating DNA fractions were removed using TURBO DNA-free Kit (Ambion, USA) following the manufacturers recommendations. The yield and quality of the resulting RNA fractions were checked on an RNA6000 chip using the 2100 Bioanalyzer system (Agilent, Santa Clara, USA).

### Double stranded copy DNA synthesis

First strand DNA was synthesized using SuperScript III Reverse transcriptase (Invitrogen, Carlsbad, USA) starting with 10 μg of RNA. For second strand DNA synthesis we used 30 units of *Escherichia coli* DNA Polymerase I (New England Biolabs, Ipswich, USA) in the presence of 2.5 units of RNAse H (Epicentre, Madison, USA), 5 units *E. coli* DNA Ligase (New England Biolabs, Ipswich, USA), Ligase buffer, DNA Polymerase I Buffer (NEB2) and 300 μM of dNTPs. Water was added to 100 μl and the mixture was incubated for 150 min at 16 °C. Subsequently 5 U of T4 DNA Polymerase was added and incubated for a further 30 min at 16 °C. The final products were cleaned using the cycle pure DNA clean up kit and stored at −20 °C.

### Sequencing

For high throughput sequencing of the cDNA, both the light and dark samples were sent to BGI (Hong-Kong). High throughput sequencing was performed using the Illumina HiSeq2000 PE100 technology yielding 231 and 222 million reads respectively of ~93 bp length. Mapping statistics can be found in Table 1. Depth of sequencing achieved from Illumina sequencing (×425-500) was sufficient to provide adequate coverage of the mRNA fraction without rRNA depletion.

### Bioinformatics

Sequence reads were pre-processed to remove low-quality bases. Reads were first mapped using Bowtie 2 software [56] against HQSB001 ribosomal operon sequences. Remaining reads were subsequently mapped to the chromosome and the plasmid sequence with the default parameters and using the pair-end strategy. SAMtools [57] were used to convert resulting data into BAM format. BamView [58] and Artemis [59] was subsequently used for the visualization of the sequence reads against the *H. walsbyi* HBSQ001 genome. Once the transcripts were mapped to the genome, the expression values of genes were calculated as the number of reads aligned over each coding DNA sequence (CDS). Gene expression values were computed using TPM normalization (transcript per million). Genes exhibiting a > 2 fold log change in TPM between two conditions or strains were considered to have differential expression. To quantify high and low expression values, we used the average median value for all conditions within both strains as a cut-off.

Functional annotation was performed by comparing predicted protein sequences against the NCBI-nr database, Pfam [60] and arCOGS [30] (cut-off E-value 10^−5^).

CAI was calculated using the program “CUSP” and “CAI” from the EMBOSS package. A subset of 40 ribosomal proteins was used to construct a reference table to which the codon usage of the genes were compared.

The transcriptome sequencing data has been submitted to NCBI SRA and is available with the bioproject number PRJNA362183 and the SRA accession numbers SRR5714110 (light sample) and SRR5714111 (dark sample).

## Additional files

Additional file 1: Growth curve of *Haloquadratum walsbyi* HBSQ001. (TIFF 1820 kb)

Additional file 2: Expression of the large halomucin gene, *hmu*, and the adjacent *bat* promotor of bacteriorhodopsin production. (TIFF 1506 kb)

Additional file 3: Full list of gene transcription levels ordered by different functional groups. (XLSX 766 kb)

Additional file 4: Polycistronic and monocistronic transcritpion in A) ATP-synthase gene cluster, B) NADH- dehydrogenase gene cluster and C) gas vesicle gene cluster. (PNG 259 kb)

## Abbreviations

arCOG: archaeal cluster of orthologous groups
ATP: adenosine triphosphate
bp: base pair
CAI: codon adaptation index (CAI)
cDNA: copy DNA
CDS: coding DNA sequence
CRISPR: clustered regularly interspaced short palindromic repeats
DHA: dihydroxyacetone
DNA: deoxyribonucleic acid
Fe-S: iron sulfide
GI: genomic island
HAS: hypersaline artificial seawater
M: Molar
MgCl2: magnesium chloride
NaCL: sodium chloride
NAD: nicotinamide adenine dinucleotide
NADH: nicotinamide adenine dinucleotide hydride
NADPH: nicotinamide adenine dinucleotide phosphate
ncRNA: non-coding RNA
nc-sRNA: non-coding small RNA
PHB: polyhydroxybutyrate
PR: proteorhodopsin
RNA: ribonucleic acid
rRNA: ribosomal RNA
S-layer: surface layer
snoRNA: small nucleolar RNAs
TCA: tricarboxylic acid
TPM: transcripts per million
tRNA: transfer RNA
UTR: untranslated but transcribed region
