# Supplementary figures and images for: Transcriptome analysis of Haloquadratum walsbyi: vanity is but the surface
Source: BMC Genomics. 2017 Jul 3;18:510. doi: 10.1186/s12864-017-3892-2 (PMC5496347; doi:10.1186/s12864-017-3892-2)

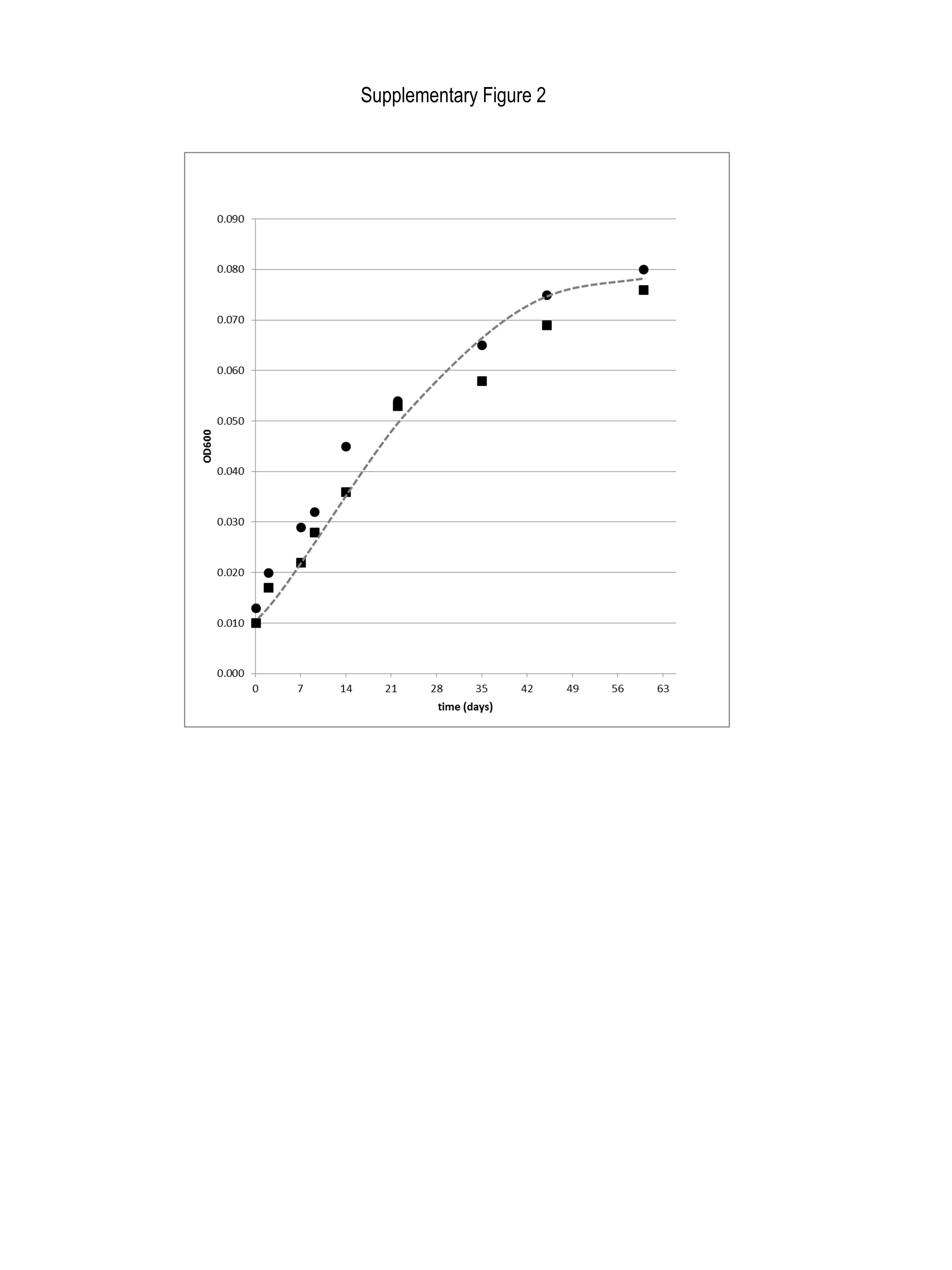

Supplement: Supplementary file 1 — Growth curve of Haloquadratum walsbyi HBSQ001. (TIFF 1820 kb) [file 12864_2017_3892_MOESM1_ESM.tif]

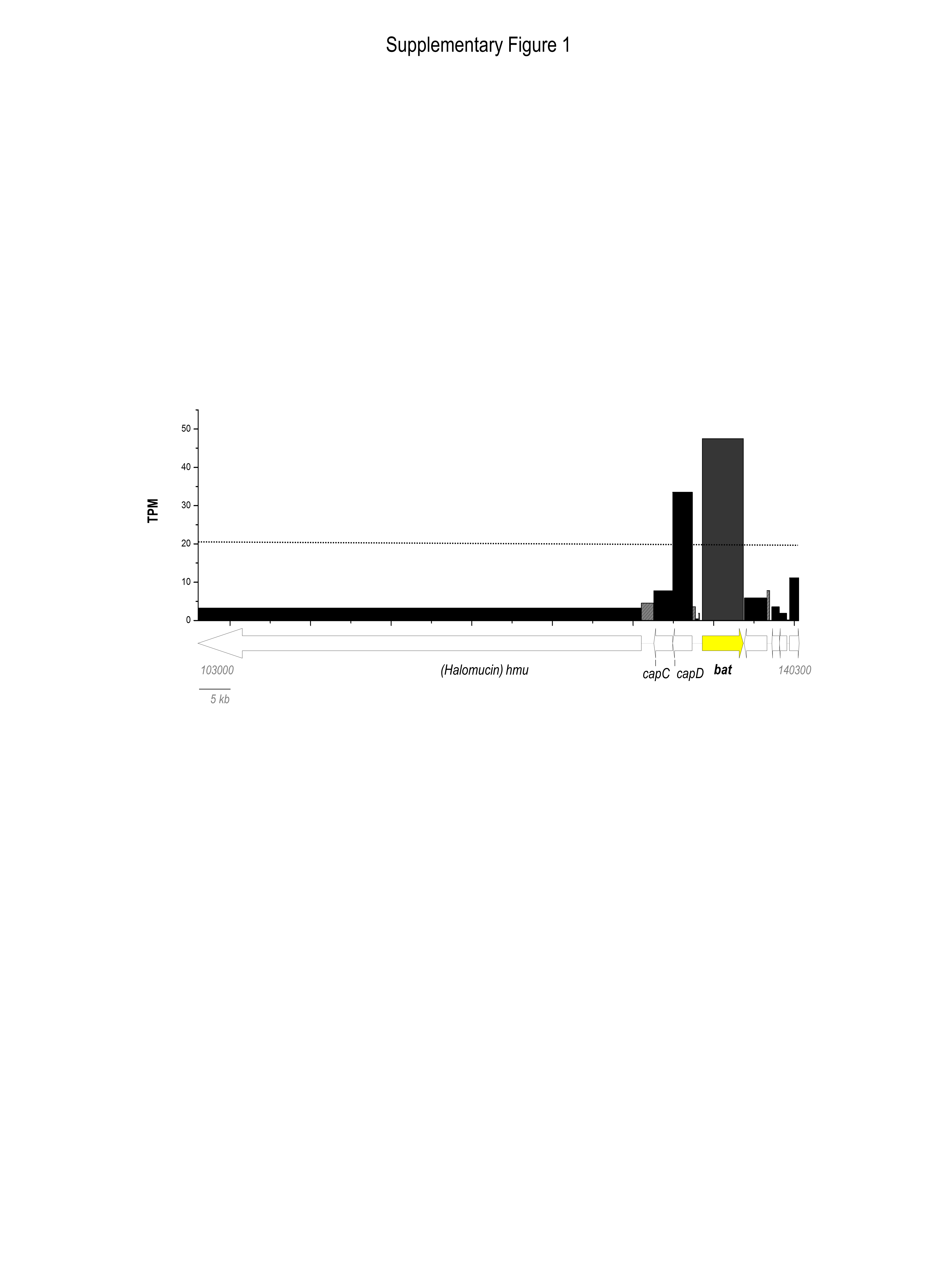

Supplement: Supplementary file 2 — Expression of the large halomucin gene, hmu, and the adjacent bat promotor of bacteriorhodopsin production. (TIFF 1506 kb) [file 12864_2017_3892_MOESM2_ESM.tif]

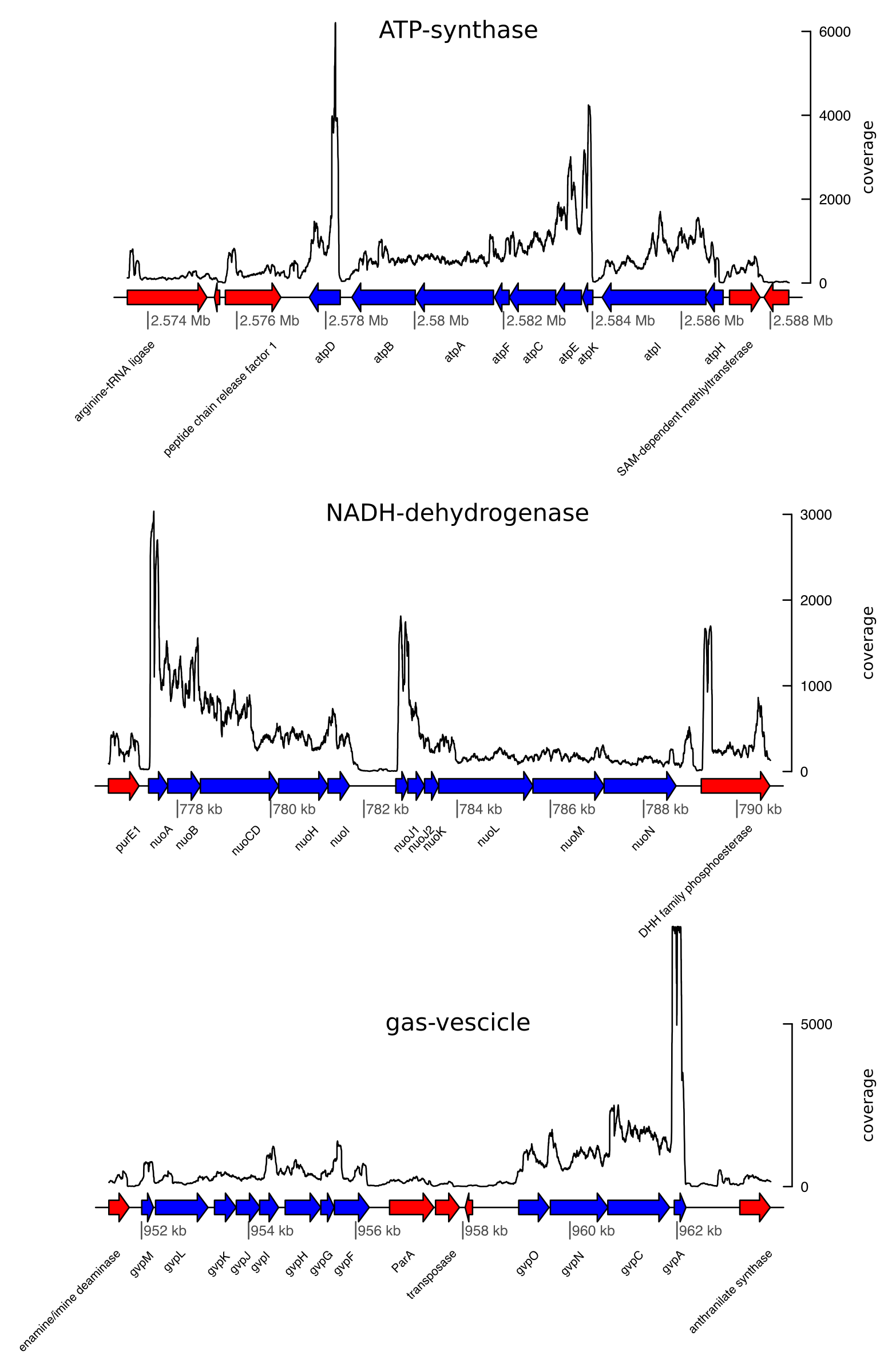

Supplement: Supplementary file 4 — Polycistronic and monocistronic transcritpion in A) ATP-synthase gene cluster, B) NADH- dehydrogenase gene cluster and C) gas vesicle gene cluster. (PNG 259 kb) [file 12864_2017_3892_MOESM4_ESM.png]
